# Towards fungal sensing skin

**DOI:** 10.1186/s40694-021-00113-8

**Published:** 2021-05-12

**Authors:** Andrew Adamatzky, Antoni Gandia, Alessandro Chiolerio

**Affiliations:** 1 Unconventional Computing Laboratory, UWE, Bristol, UK; 2 Mogu S.r.l., Inarzo, Italy; 3 Center for Sustainable Future Technologies, Istituto Italiano di Tecnologia, Torino, Italy

**Keywords:** Fungi, Biomaterials, Sensing, Sensorial fusion, Soft robotics

## Abstract

A fungal skin is a thin flexible sheet of a living homogeneous mycelium made by a filamentous fungus. The skin could be used in future living architectures of adaptive buildings and as a sensing living skin for soft self-growing/adaptive robots. In experimental laboratory studies we demonstrate that the fungal skin is capable for recognising mechanical and optical stimulation. The skin reacts differently to loading of a weight, removal of the weight, and switching illumination on and off. These are the first experimental evidences that fungal materials can be used not only as mechanical ‘skeletons’ in architecture and robotics but also as intelligent skins capable for recognition of external stimuli and sensorial fusion.

## Background

Flexible electronics, especially electronic skins [1–3] is amongst the most rapidly growing and promising fields of novel and emergent hardware. The electronic skins are made of flexible materials where electronics capable of tactile sensing [4–7] are embedded. The electronic skins are capable of low level perception [8, 9] and could be developed as autonomous adaptive devices [10]. Typical designs of electronic skins include thin-film transistor and pressure sensors integrated in a plastic substrate [11], micro-patterned polydimethylsiloxane with carbon nanotube ultra-thin films [12, 13], a large-area film synthesised by sulfurisation of a tungsten film [14], multilayered graphene [15], platinum ribbons [3], Polyethylene terephthalate (PET) based silver electrodes [16], digitally printed hybrid electrodes for electromyographic recording [17] or for piezoresistive pressure sensing [18], or channels filled with conductive polymer [19].

**Fig. 1:**
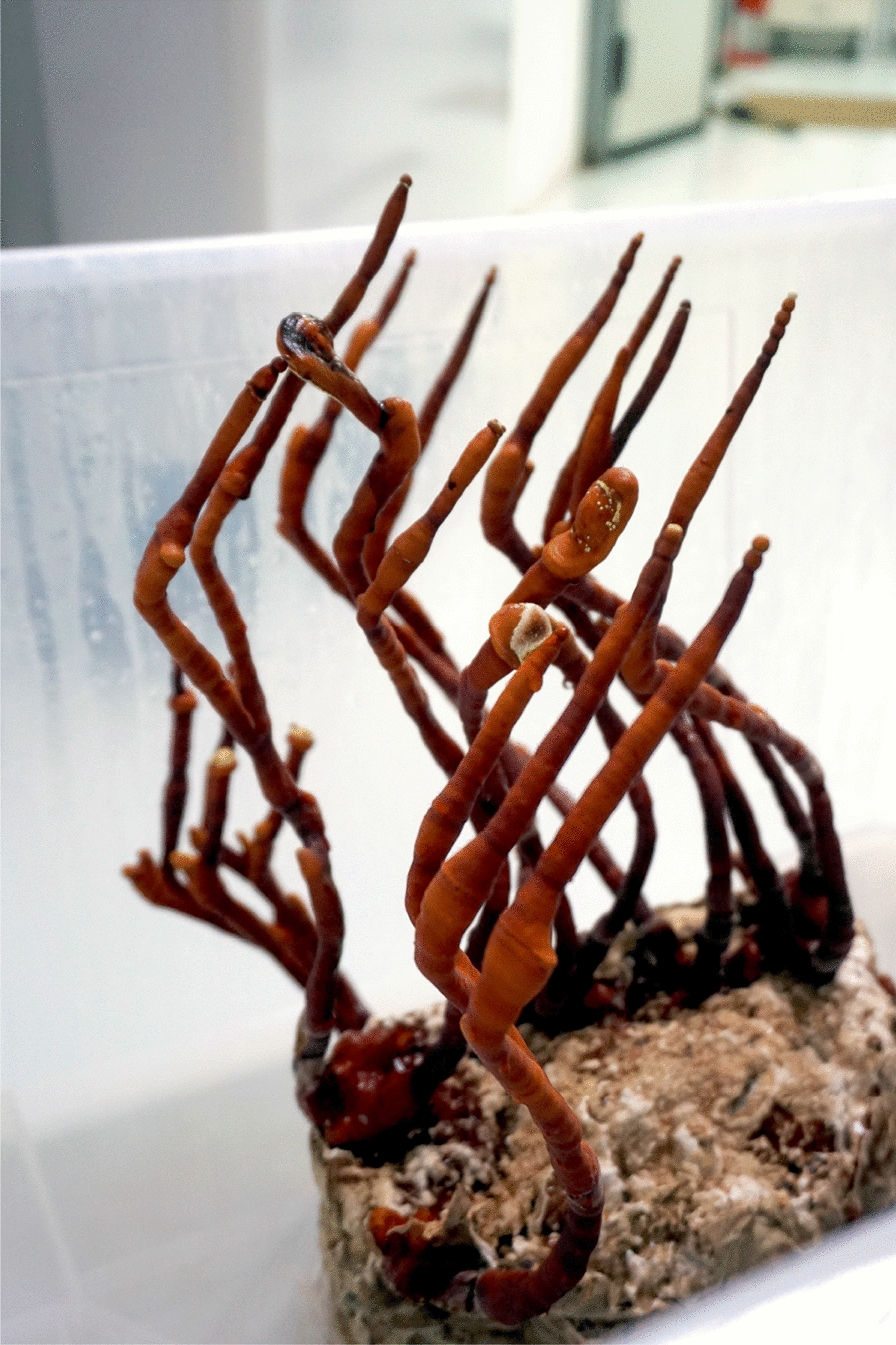
Phototropism is one of the leading guiding factors in the formation of basidiocarps in *Ganoderma* spp.

Whilst the existing designs and implementations are highly impactful, the prototypes of electronic skins lack a capacity to self-repair and grow. Such properties are useful, and could be necessary, when an electronic skin is used in e.g. unconventional living architecture [20], soft and self-growing robots [21–24] and development of intelligent materials from fungi [25–28]. Based on our previous experience with designing tactile, colour sensors from slime mould *Physarum polycephalum* [29–31] and our recent results on fungal electrical activity [32–34], as well as following previously demonstrated thigmotropic and phototropic response (Fig. 1) in higher fungi [35], we decided to propose a thin layer of homogeneous mycelium of the trimitic polypore species *Ganoderma resinaceum* as a live electronic skin and thus investigate its potential to sense and respond to tactile and optical stimuli. We call the fungal substrate, used in present paper, ‘fungal skin’ due to its overall appearance and physical feeling. In fact, several species of fungi have been proposed as literal skin substitutes and tested in wound healing [36–41].

The paper is structured as follows. Patterns of electrical activity of the fungal skin are analysed in “Results” section. Results are considered in a wider context and directions of future studies are outlined in “Discussion” section. The protocol for growing the fungal skin and the methods of electrical activity recording are described in “Methods” section.

## Results

Endogenous electrical activity of the fungal material is polymorphic. Low and high frequency oscillations patterns can emerge intermittently. A train of four spikes in Fig. 4c is an example of low frequency oscillations. By measuring the electrical response with multiple electrodes, positioned along coordinated axes like row and columns of a matrix, and connecting them to a differential operational amplifier, it is possible to exclude singularities and enhance coordinated responses, which is indicated as a filtering procedure to exclude endogenous polymorphic activity.

**Fig. 2:**
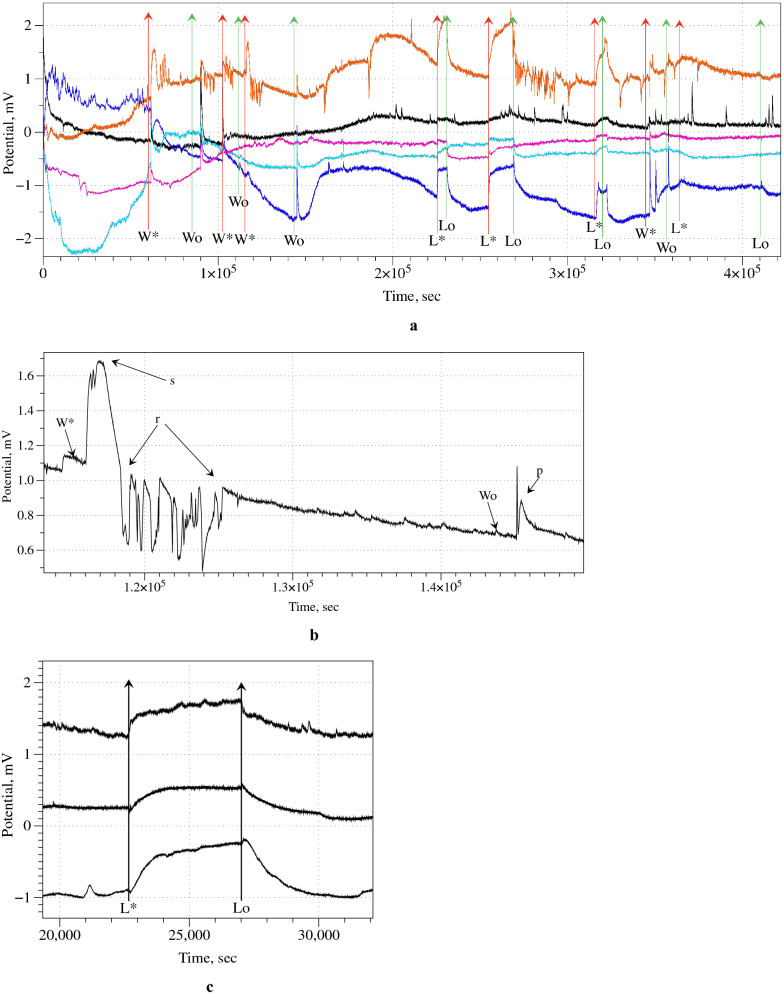
Fungal skin response to mechanical and optical stimulation. **a** Exemplar recording of fungal skin electrical activity under tactile and optical stimulation. Moments of applying and removing a weight are shown as ‘W*’ and ‘Wo’ and switching light ON and OFF as ‘L*’ and ‘Lo’. **b** Exemplar response to mechanical stimulation. Moments of applying and removing a weight are shown as ‘W*’ and ‘Wo’. High-amplitude response is labelled ‘s’. This response is followed by a train of spikes ‘r’. A response to the removal of the weight is labelled ‘p’. **c** Exemplar response of fungal skin to illumination, recorded on three pairs of differential electrodes. ‘L*’ indicates illumination is applied, ‘Lo’ illumination is switched off

Electrical responses to tactile loading and illumination are distinctive and can be easily recognized from endogenous activity. An example of several rounds of stimulation is shown in Fig. 2a. The fungal skin responds to loading of a weight with a high-amplitude wide spike of electrical potential sometimes followed by a train of high-frequency spikes. The skin also responds to removal of the weight by a high-amplitude spike of electrical potential.

An exemplar response to loading and removal of weight is shown in Fig. 2b. The parameters of the fungal skin responses to the weight being placed on the skin are the following. An average delay of the response (the time from weight application to a peak of the high-amplitude spike) is 911.4 s ($\sigma =1280.1$, minimum 25 s and maximum 3200 s). An average amplitude of the response spike (marked ‘s’ in the example Fig. 2b) is 0.4 mV ($\sigma =0.2$, minimum 0.1 mV and maximum 0.8 mV). An average width of the response spike is 1261.8 s ($\sigma =1420.3$, minimum 199 s and maximum 4080 s), meaning that the average energy consumed per current unit, associated to the response, is approximately 0.5 J/A. A train of spikes (marked ‘r’ in the example Fig. 2b), if any, following the response spike usually has 4 or 5 spikes. The fungal skin responds to removal of the weight (the response is marked ‘p’ in the example Fig. 2b) with a spike which average amplitude is 0.4 mV ($\sigma =0.2$, minimum 0.2 mV and maximum 0.85 mV). Amplitudes are less indicative than frequencies because an amplitude depends on the position of electrodes with regards to propagating wave of excitation. An average width of the spike is 774 s ($\sigma =733.1$, minimum 100 s and maximum 2000 s. A response of the fungal skin to removal of the weight was not observed in circa 20% of differential electrode pairs. The average response time is 385.5 s ($\sigma =693.3\, \text{s}$, minimum 77 s and maximum 1800 s). By taking into account inter-electrode distance it could be possible to weigh temporal delays and further strengthen the rejection circuits based on operational amplifiers, as per above suggestion to discard endogenous activity.

The response of the fungal skin to illumination is manifested in the raising of the baseline potential, as illustrated in the exemplar recordings in Fig. 2c. In contrast to mechanical stimulation response the response-to-illumination spike does not subside but the electrical potential stays raised until illumination is switched off. An average amplitude of the response is 0.61 mV ($\sigma =0.27$, minimum 0.2 mV and maximum 1 mV). The raise of the potential starts immediately after the illumination is switched on. The potential saturation time is 2960 s in average ($\sigma =2201$, minimum 879 s and maximum 9530 s); the potential relaxation time is 8700 s in average ($\sigma =4500$, minimum 962 s and maximum 24790 s).

In the case of illumination it is particularly easy to imagine how effective a rejection stage could be, since all the responses are well synchronized (Fig. 2c).

## Discussion

**Fig. 3:**
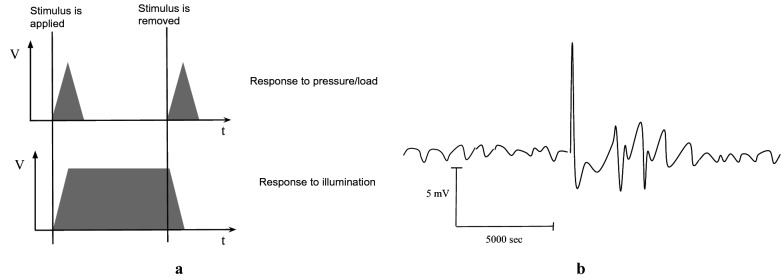
**a** A scheme of the fungal skin responses to mechanical load and optical stimulations. **b** Slime mould *P. polycephalum* response to application of 0.01 g glass capillary tube. Redrawn from [30]

We demonstrated that a thin sheet of homogeneous living mycelium of *Ganoderma resinaceum*, which we named ‘fungal skin’, shows pronounced electrical responses to mechanical and optical stimulation. Can we differentiate between the fungal skin’s response to mechanical and optical stimulation? Definitely, see Fig. 3a. The fungal skin responds to mechanical stimulation with a 15 min spike of electrical potential, which diminishes even if the applied pressure on the skin remains. The skin responds to optical stimulation by raising its electrical potential and keeping it raised till the light is switched off.

Can we differentiate the responses to loading and removal of the weight? Yes. Whilst amplitudes of ‘loading’ and ‘removal’ spikes are the same (0.4 mV in average) the fungal skin average reaction time to removal of the weight is 2.4 times shorter than the reaction to loading of the weight (385 s versus 911 s). Also ‘loading’ spikes are 1.6 times wider than ‘removal’ spikes (1261 s versus 774 s).

Fungal skin response to weight application is, in some cases, esp. Fig. 2b, similar to response of slime mould to application of the light weight [30]. The following events are observed (Fig. 3b): oscillatory activity before stimulation, immediate response to stimulation, prolonged response to stimulation as a train of high-amplitude spikes, return to normal oscillatory activity. This might indicate some universal principles of sensing and information processing in fungi and slime moulds.

The sensing fungal skin proposed has a range of advantages comparing to other living sensing materials, e.g. slime mould sensors [29–31] electronic sensors with living cell components [42], chemical sensors using living taste, olfactory, and neural cells and tissues [43] and tactile sensor from living cell culture [44]. The advantages are low production costs, simple maintenance and durability. The last but not least advantage is scalability: a fungal skin patch can be as small as few milimeters or it can be grown to several metres in size.

In future studies we will aim to answer the following questions. Would it be possible to infer a weight of the load applied to the fungal skin from patterns of its electrical activity? Would the fungal skin indicate directionality of the load movement by its spiking activity? Would it be possible to locate the position of the weight within the fungal network? Would it be possible to map a spectrum of the light applied to the skin onto patterns of the skin’s electrical activity?

## Methods

Potato dextrose agar (PDA), malt extract agar (MEA) and malt extract (ME) were purchased from Sigma-Aldrich (USA). The *Ganoderma resinaceum* culture used in this experiment was obtained from a wild basidiocarp found at the shores of *Lago di Varese*, Lombardy (Italy) in 2018 and maintained in alternate PDA and MEA slants at MOGU S.r.l. for the last 3 years at 4 °C under the collection code 019-18.

**Fig. 4:**
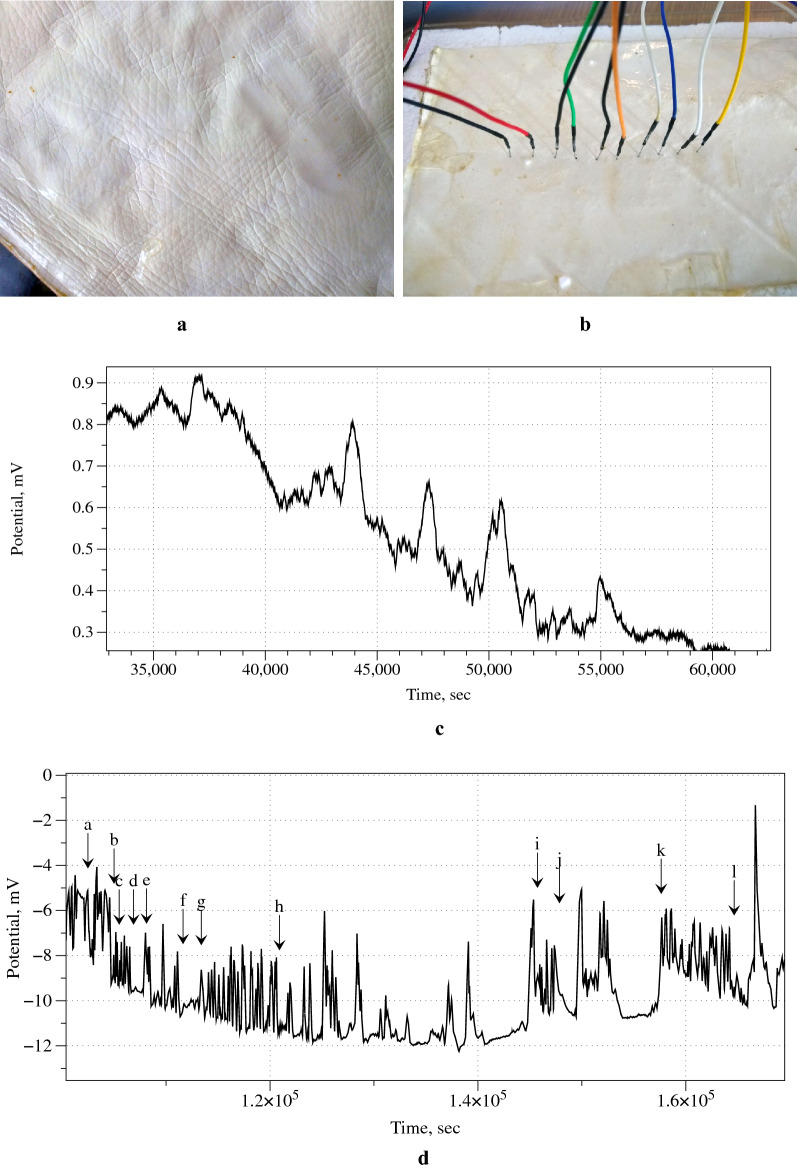
Recording of electrical activity of fungal skin. **a** Close-up texture detail of a fungal skin. **b** A photograph of electrodes inserted into the fungal skin. **c** Train of three low-frequency spikes, average width of spikes there is 1500 s, a distance between spike peaks is 3000 s and average amplitude is 0.2 mV. **d** Example of several train of high-frequency spikes. Each train $T_{xy}=(A_{xy}, W_{xy}, P_{xy})$ is characterised by average amplitude of spikes $A_{xy}$ mV, width of spikes $W_{xy}$ sec and average distance between neighbouring spikes’ peaks $P_{xy}$ sec: $T_{ab}=(2.6, 245, 300)$, $T_{cd}=(1.7, 160, 220)$, $T_{ef}=(1.6, 340, 340)$, $T_{gh}=(2.5, 240, 350)$, $T_{ij}=(2.5, 220, 590)$, $T_{kl}=(2.6, 290, 440)$

The fungal skin was prepared as follows. *G. resinaceum* was grown on MEA plates and a healthy mycelium plug was inoculated into an Erlenmeyer flask containing 200 mL of 2% ME broth (MEB). The liquid culture flask was then incubated in a rotary shaker at 200 rpm and $28~^{\circ }\text {C}$ for 5 days. Subsequently, this liquid culture was homogenised for 1 min at max. speed in a sterile 1 L Waring laboratory blender (USA) containing 400 mL of fresh MEB, the resulting 600 mL of living slurry were then poured into a 35 by 35 cm static fermentation tray. The slurry was let to incubate undisturbed for 15 days to allow the fungal hyphae to inter-mesh and form a floating mat or skin of fungal mycelium. Finally, a living fungal skin circa 1.5 mm thick was harvested (see texture of the skin in Fig. 4a), washed in sterile demineralised water, cut to the size 23 cm by 11 cm and placed onto a polyurethane base to keep electrodes stable during the electrical characterisation steps (Fig. 4b).

The electrical activity of the skin was measured as follows. We used iridium-coated stainless steel sub-dermal needle electrodes (Spes Medica S.r.l., Italy), with twisted cables. The pairs of electrode were inserted in the fungal skin as shown (Fig. 4b): the first placed in position $2 \times 5\,\text {cm}$ from a vertex, the following placed at 1 cm distance each. In each pair we recorded a difference in electrical potential between the electrodes. We used ADC-24 (Pico Technology, UK) high-resolution data logger with a 24-bit Analog to Digital converter, galvanic isolation and software-selectable sample rates. We recorded electrical activity with a frequency of one sample per second. We set the acquisition voltage range to 156 mV with an offset accuracy of $9\,\upmu \text {V}$ to maintain a gain error of 0.1%. For mechanical stimulation with 30 g nylon cylinder placed at 3 cm from the long edge and 3 from the electrodes, and aligned with electrode number 5, contact area with the fungal skin was circa 35 mm disc. For optical stimulation we used an aquarium light, array of LEDs, 36 white LEDs and 12 blue LEDs, 18 W, illumination on the fungal skin was 0.3 Lux.

## Data Availability

The datasets used and/or analysed during the current study are available from the corresponding author on reasonable request.
